# Treatment Outcomes of Squamous Cell Carcinoma of the Soft Palate and the Prognostic Significance of HPV/p16 Status

**DOI:** 10.1002/hed.28143

**Published:** 2025-03-27

**Authors:** Meng‐hua Li, Xing Zhang, Feng‐jiao Li, Xian‐lu Gao, Shi‐da Yan, Qiao‐hong Lin, Xi‐yuan Li, Jian Meng, Ying Zhang, Shi‐ting Zhang, Shu‐wei Chen, Ming Song

**Affiliations:** ^1^ Department of Head and Neck Surgery Sun Yat‐Sen University Cancer Center Guangzhou China; ^2^ State Key Laboratory of Oncology in South China Guangzhou China; ^3^ Guangdong Provincial Clinical Research Center for Cancer Guangzhou China; ^4^ Department of Operation Theater Services Sun Yat‐Sen University Cancer Center Guangzhou China

**Keywords:** HPV, oropharyngeal cancer, prognosis, radiotherapy, surgery, treatment

## Abstract

**Background:**

Squamous cell carcinoma of the soft palate (SCCSP) represents a rare subtype of oropharyngeal cancer. This study aims to evaluate the treatment outcomes of SCCSP and to assess the prognostic significance of HPV status.

**Methods:**

Patients diagnosed with SCCSP between January 1981 and December 2021 were collected. Survival outcomes were compared.

**Results:**

In univariate analysis, primary surgery resulted in superior progression‐free survival (PFS), overall survival (OS), and disease‐specific survival (DSS) compared with definitive radiotherapy (*p* < 0.05). Furthermore, multivariate analysis revealed that primary surgery independently correlated with superior PFS (HR = 0.37, *p* = 0.002), OS (HR = 0.55, *p* = 0.012), and DSS (HR = 0.45, *p* = 0.020) in early‐stage SCCSPs. Additionally, no significant prognostic differences were observed between HPV/p16 positive and HPV/p16 negative SCCSPs (*p* > 0.05).

**Conclusion:**

Surgery yields superior oncological outcomes for early‐stage SCCSP patients. HPV status does not demonstrate prognostic significance in SCCSP.

## 1 Introduction

The incidence of oropharyngeal squamous cell carcinoma (OPSCC) has risen dramatically on a global scale, largely attributed to the increasing prevalence of human papillomavirus (HPV) within the population [1]. The oropharynx is comprised of four primary subsites: the tonsils, the base of tongue, the soft palate, and the posterior pharyngeal walls. Squamous cell carcinoma of the soft palate (SCCSP) is a relatively rare entity, accounting for approximately 10% of all OPSCCs [2]. In comparison to tonsillar‐related OPSCCs (squamous cell carcinomas of the tonsils and the base of tongue), SCCSP is linked to a poorer prognosis, with a reported 5‐year survival rate of only about 40% [3, 4].

The soft palate is composed of a stratified squamous epithelium that lacks a keratinized layer, similar to the epithelium found in the oral cavity. In contrast to the tonsillar‐related areas in the oropharynx, lymphoid tissue is not abundantly present in the soft palate [5]. Previous research has indicated that HPV‐positive OPSCC demonstrates a high responsiveness to treatment and is associated with a favorable prognosis. However, the occurrence of HPV‐positive disease in the soft palate is relatively infrequent [6]. Furthermore, the prognostic implications of HPV status in SCCSP remain ambiguous due to its rarity. Given that SCCSPs are typically located superficially and can be readily visualized during examination, they can often be diagnosed at an early stage. The management of SCCSP is still a subject of controversy, with ongoing debates regarding treatment modalities, including surgery and radiotherapy [7]. Additionally, literature specifically addressing SCCSP is limited, and there is a scarcity of studies featuring large sample sizes and long‐term outcome data.

In this study, we aim to evaluate the treatment outcomes of patients with SCCSP and to assess the influence of HPV status on prognosis within a large tertiary cancer center located in South China.

## 2 Patients and Methods

### 2.1 Patient Identification and Data Collection

We retrospectively included consecutive patients diagnosed with SCCSP between January 1981 and December 2021 at the Sun Yat‐sen University Cancer Center, a tertiary referral center located in South China. Clinical, pathological, and demographic information was extracted from the medical records of eligible patients. The inclusion criteria were as follows: (i) histologically confirmed squamous cell carcinoma located in the soft palate or uvula; (ii) a minimum of 2 years of clinical follow‐up for patients who did not experience tumor progression or who were alive at the last follow‐up. Patients with distant metastatic disease or other uncontrolled malignancies at the time of initial presentation were excluded from the study. This study was approved by our hospital's Institutional Review Board, and the ethics committee review specifically waived the need for informed consent.

HPV/p16 positivity was determined through the detection of high‐risk HPV types utilizing the real‐time polymerase chain reaction (RT‐PCR) method or by positive immunohistochemistry for p16, characterized by diffuse and strong cytoplasmic and nuclear staining in more than 70% of tumor cells [8]. In this study, tumor staging was conducted in accordance with the seventh edition of the American Joint Committee on Cancer (AJCC) staging guidelines for oropharyngeal cancers. The eighth edition of the AJCC staging guidelines was not applied due to the unknown HPV/p16 status in a significant proportion of the patient cohort.

### 2.2 Treatment Groups

Patients were evaluated by our multidisciplinary head and neck cancer board before the commencement of treatment. The initial treatment approach was categorized into two groups: the primary surgery group and the definitive radiotherapy group.

The primary surgery cohort comprised patients who underwent surgery with curative intent. Unilateral or bilateral therapeutic neck dissection was conducted for patients with clinically suspected lymph node metastasis, while prophylactic lymph node dissection was not actively performed for patients with cN0 tumors.

The definitive radiotherapy cohort comprised patients who underwent radiotherapy, either alone or in conjunction with concurrent therapy, which may or may not have included induction chemotherapy. The concurrent therapy regimen encompassed platinum‐based therapies and Nimotuzumab, or was combined with oral fluorouracil (capecitabine). Induction chemotherapy involved platinum‐based therapies in combination with paclitaxel‐based therapies, or was administered alongside either 5‐fluorouracil or oral fluorouracil (capecitabine).

### 2.3 Outcomes and Statistical Analysis

Differences in patient characteristics concerning treatment regimens and HPV/p16 status were analyzed using the Student's *t*‐test, Fisher's exact test, Mann–Whitney *U* test, and chi‐squared test. The survival endpoints included progression‐free survival (PFS), overall survival (OS), and disease‐specific survival (DSS). PFS was defined as the interval from the date of initial treatment to the date of tumor progression, which encompassed local recurrence, lymph node metastasis, distant metastasis, or death, with surviving patients without evidence of disease being censored at the last follow‐up. OS was calculated from the date of diagnosis to the date of death from any cause, with surviving patients censored at the last follow‐up. DSS was calculated from the date of diagnosis to the date of death specifically from SCCSP, with patients who did not experience SCCSP‐related death being censored at the date of the last follow‐up or at the time of non‐SCCSP‐related death. Survival time was estimated using the Kaplan–Meier method and compared using the log‐rank test for significance. The Cox proportional regression model was used for multivariate analysis. All tests were two‐sided. A *p*‐value of less than 0.05 was considered statistically significant. All data were analyzed using SPSS 26 software (SPSS, Chicago, IL).

## 3 Results

### 3.1 Characteristics of Patients

A total of 256 consecutive patients were enrolled in this study. All patients were ethnic Chinese. The mean age at diagnosis was 57.63 years, with a notable male predominance observed (91.4%). A majority of the patients (68.8%) were smokers, and approximately half of these individuals (49.2%) were drinkers. Twenty‐two patients had a prior history of cancer, with 14 cases located in the head and neck region and 8 cases in other anatomical sites. The baseline characteristics of the study population are summarized in Table 1.

**TABLE 1:** Patient and tumor characteristics.

Variable	Number (%)	Primary surgery, *n* = 99 (%)	Definitive RT, *n* = 148 (%)	*p*	HPV/p16+, *n* = 25 (%)	HPV/p16−, *n* = 62 (%)	*p*
Age, y	57.63 (9.44)	57.32 (8.67)	57.57 (9.87)	0.156	55.20 (9.03)	56.87 (9.03)	0.439
Gender				0.218			0.348
Male	234 (91.4)	87 (87.9)	138 (93.2)		22 (88.0)	59 (95.2)	
Female	22 (8.6)	12 (12.1)	10 (6.8)		3 (12.0)	3 (4.8)	
Smoker				0.075			1
Negative	80 (31.2)	39 (39.4)	38 (25.7)		7 (28.0)	16 (25.8)	
Positive	176 (68.8)	60 (60.6)	110 (74.3)		18 (72.0)	46 (74.2)	
Drinker				0.296			0.058
Negative	130 (50.8)	56 (56.6)	69 (46.6)		7 (28.0)	30 (48.4)	
Positive	126 (49.2)	43 (43.4)	79 (53.4)		18 (72.0)	32 (51.6)	
Previous cancer				0.034			1
No	234 (91.4)	85 (85.9)	141 (95.3)		23 (92.0)	57 (91.9)	
Yes	22 (8.6)	14 (14.1)	7 (4.7)		2 (8.0)	5 (8.1)	
HPV/p16 status				0.004			
Negative	62 (71.3)	44 (84.6)	17 (51.5)				
Positive	25 (28.7)	8 (15.4)	16 (48.5)				
Unknown	169	47	115				
RN involved	24 (9.4)	0	20 (13.5)	< 0.001	4 (16.0)	5 (8.1)	0.272
cT stage				< 0.001			0.796
T1	81 (31.6)	35 (35.4)	45 (30.4)		8 (32.0)	19 (30.6)	
T2	113 (44.1)	56 (56.6)	56 (37.8)		11 (44.0)	29 (46.8)	
T3	29 (11.3)	4 (4.0)	22 (14.9)		2 (8.0)	8 (12.9)	
T4	33 (12.9)	4 (4.0)	25 (16.9)		4 (16.0)	6 (9.7)	
cN stage				0.652			0.108
N0	140 (54.7)	60 (60.6)	79 (53.4)		15 (60.0)	34 (54.8)	
N1	31 (13.3)	11 (11.1)	23 (15.5)		3 (12.0)	8 (12.9)	
N2	74 (28.9)	25 (25.3)	42 (28.4)		5 (20.0)	20 (32.3)	
N3	8 (3.1)	3 (3.0)	4 (2.7)		2 (8.0)	0	
Overall c‐stage				0.381			0.111
I	60 (23.4)	27 (27.3)	33 (22.3)		7 (28.0)	15 (24.2)	
II	62 (24.2)	29 (29.3)	32 (21.6)		6 (24.0)	14 (22.6)	
III	42 (16.4)	14 (14.1)	28 (18.9)		6 (24.0)	12 (19.4)	
IVa	79 (30.9)	24 (24.2)	48 (32.4)		4 (16.0)	21 (33.9)	
IVb	13 (5.1)	5 (5.1)	7 (4.7)		2 (8.0)	0	
Treatment							0.004
Primary surgery	99 (38.7)				8 (32.0)	44 (71.0)	
Definitive RT	148 (57.8)				16 (64.0)	17 (27.4)	
RT/CRT	118				12	10	
ICT + RT/CRT	30				4	7	
Palliative	9 (3.5)				1 (4.0)	1 (1.6)	

*Note*: Continuous values are presented as numbers or mean ± SD.

Abbreviations: CRT, chemoradiotherapy; c‐stage, clinical stage; ICT, induction chemotherapy; RN, Retropharyngeal nodes; RT, radiotherapy.

The majority of patients initially presented with early T‐stage (T1‐2; *n* = 194, 75.7%) and early N‐stage diseases (N0‐1; *n* = 171, 68.0%). Additionally, 141 patients (55.1%) were classified as having overall early‐stage diseases (T1‐2 N0‐1 M0). Among the cohort, 87 patients underwent HPV/p16 testing, of which 25 patients (28.7%) were identified as HPV/p16 positive. When compared to HPV/p16 negative patients, those who were HPV/p16 positive were significantly more likely to receive definitive radiotherapy (64.0% vs. 27.4%, *p* = 0.004).

### 3.2 Treatment Characteristics

Among the total patient cohort, 99 individuals (38.7%) underwent primary surgery, of which 36 subsequently received postoperative radiotherapy (PORT). Additionally, 148 patients (57.8%) were treated with definitive radiotherapy, with 30 of these patients having received induction chemotherapy prior to the radiotherapy. Induction chemotherapy was administered to patients presenting with clinical T2‐4 stage tumors. Nine patients received palliative treatment due to advanced disease; the palliative treatment regimens included platinum‐based therapies, paclitaxel‐based therapies, or combinations thereof with either 5‐fluorouracil or oral fluorouracil (capecitabine) chemotherapy.

In comparison to patients who underwent primary surgery, those in the definitive radiotherapy group exhibited a lower likelihood of having a prior history of cancer (*p* = 0.034). Conversely, they demonstrated a higher likelihood of presenting with HPV/p16 positive disease (*p* = 0.004), retropharyngeal lymph node involvement (*p* < 0.001), and advanced clinical T‐stage disease (*p* < 0.001).

## 4 Survival Analysis

### 4.1 Oncological Outcomes Concerning Various Treatment Groups

**FIGURE 1:**
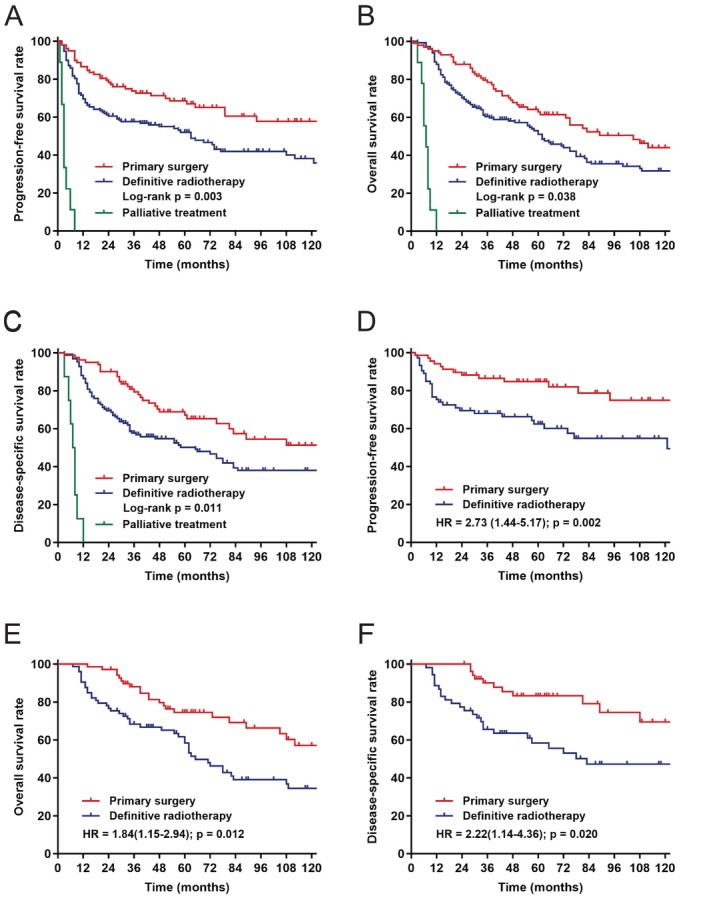
Kaplan–Meier survival curves stratified by treatment group. (A) Kaplan–Meier curve for PFS in the whole cohort stratified by treatment group. (B) Kaplan–Meier curve for OS in the whole cohort stratified by treatment group. (C) Kaplan–Meier curve for DSS in the whole cohort stratified by treatment group. (D) Kaplan–Meier curve for PFS in early‐stage SCCSPs stratified by treatment group. (E) Kaplan–Meier curve for OS in early‐stage SCCSPs stratified by treatment group. (F) Kaplan–Meier curve for DSS in early‐stage SCCSPs stratified by treatment group. [Color figure can be viewed at wileyonlinelibrary.com]

The median follow‐up duration for all patients was 52 months (range: 4–504 months), while for surviving patients, it was 68 months (range: 25–504 months). As of the last follow‐up date, tumor progression was observed in 124 patients (48.4%), and 156 patients (60.9%) had died, including 112 patients (43.8%) who succumbed to disease‐specific causes. The 5‐year PFS, OS, and DSS for the entire cohort were 56.8%, 53.8%, and 61.4%, respectively. When analyzed by treatment modality, the 5‐year PFS, OS, and DSS rates for patients who underwent primary surgery compared to those who received definitive radiotherapy were 68.6% versus 52.0% (*p* = 0.003), 62.8% versus 52.8% (*p* = 0.038), and 72.5% versus 57.4% (*p* = 0.011), respectively. The Kaplan–Meier survival curves are presented in Figure 1.

In the primary surgery cohort, univariate analysis indicated that patients who underwent PORT experienced significantly poorer PFS (5‐year PFS: 50.9% vs. 79.5%, *p* = 0.001), OS (5‐year OS: 51.3% vs. 69.5%, *p* = 0.048), and DSS (5‐year DSS: 57.0% vs. 82.0%, *p* = 0.002) in comparison to those who did not receive PORT (see Table 2).

**TABLE 2:** Univariate and multivariate analysis for the entire cohort.

Variable	Univariate analysis		Multivariate analysis (PFS)		Univariate analysis		Multivariate analysis (OS)		Univariate analysis		Multivariate analysis (DSS)	
	5 years PFS (%)	*p*	HR (95% CI)	*p*	5 year OS (%)	*p*	HR (95% CI)	*p*	5 year DSS (%)	*p*	HR (95% CI)	*p*
whole cohort	56.8				53.8				61.4			
Age, y		0.030	1.03 (1.01–1.05)	0.004		< 0.001	1.04 (1.02–1.06)	< 0.001		< 0.001	1.04 (1.02–1.06)	< 0.001
Gender		0.495				0.484				0.255		
Male	55.7				53.2				60.0			
Female	67.9				62.9				76.5			
Smoker		0.258				0.135				0.231		
Negative	61.0				57.5				61.9			
Positive	54.7				52.6				61.2			
Drinker		0.768				0.160				0.415		
Negative	57.5				56.3				61.4			
Positive	56.0				52.0				61.4			
Previous cancer		0.570				0.911				0.561		
No	62.9				54.3				61.4			
Yes	56.2				52.4				62.9			
RN involved		0.339				0.812				0.714		
No	56.5				54.1				61.9			
Yes	57.8				50.8				55.9			
HPV/p16 status		0.662				0.460				0.839		
Negative	66.8				58.4				72.5			
Positive	67.8				72.8				72.8			
Unknown												
cT stage		< 0.001		0.006		< 0.001		0.073		< 0.001		0.025
T1‐2	63.5		1.00		59.2		1.00		67.8		1.00	
T3‐4	35.4		1.76 (1.18–2.64)		37.5		1.43 (0.97–2.10)		40.6		1.65 (1.07–2.54)	
cN stage		< 0.001		0.001		0.002		0.009		< 0.001		< 0.001
N0‐1	65.2		1.00		60.9		1.00		69.9		1.00	
N2‐3	37.9		1.93 (1.31–2.85)		38.9		1.62 (1.13–2.31)		42.2		2.09 (1.39–3.13)	
Overall c‐stage												
I‐III	68.5	Ref			64.1	Ref			74.4	Ref		
IV	35.2	< 0.001			35.4	< 0.001			38.3	< 0.001		
Treatment												
Definitive RT	52.0	Ref	1	Ref	52.8	Ref	1	Ref	57.4	Ref	1	Ref
Primary surgery	68.6	0.003	0.68 (0.45–1.04)	0.080	62.8	0.038	0.78 (0.54–1.12)	0.172	72.5	0.011	0.71 (0.46–1.11)	0.130
Palliative	0	< 0.001	10.29 (4.16–25.45)	< 0.001	0	< 0.001	27.80 (10.65–72.59)	< 0.001	0	< 0.001	28.66 (10.13–81.06)	< 0.001

Abbreviations: CI, confidence interval; c‐stage, clinical stage; HR, hazard ratio; ICT, induction therapy; RN, Retropharyngeal nodes; RT, radiotherapy.

In the definitive radiotherapy cohort, patients who underwent induction therapy did not demonstrate a statistically significant enhancement in PFS (*p* = 0.296), OS (*p* = 0.362), or DSS (*p* = 0.224). Additionally, we evaluated the prognostic implications of salvage surgery within this cohort. A total of 25 patients underwent salvage surgery due to residual locoregional disease or progression of locoregional disease. The results indicated that salvage surgery was associated with a statistically significant improvement in 5‐year OS (74.3%, *p* = 0.023) and a near statistically significant enhancement in 5‐year DSS (79.2%, *p* = 0.068).

In the multivariable analysis, no statistically significant differences were observed in PFS (*p* = 0.080), OS (*p* = 0.172), or DSS (*p* = 0.130) between patients who underwent primary surgery and those who received definitive radiotherapy (see Table 2).

### 4.2 Treatment Outcomes for Early‐Stage SCCSPs

**FIGURE 2:**
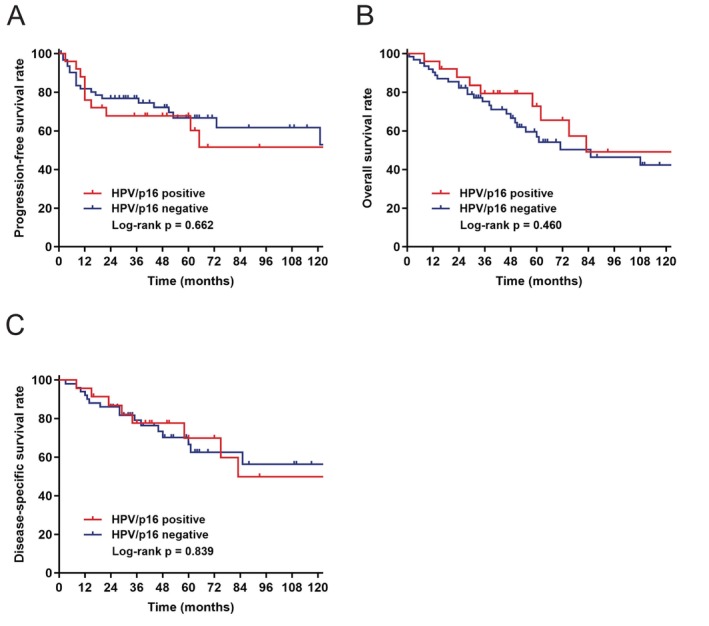
Kaplan–Meier survival curves stratified by HPV/p16 status. (A) Kaplan–Meier curve for PFS stratified by HPV/p16 status. (B) Kaplan–Meier curve for OS stratified by HPV/p16 status. (C) Kaplan–Meier curve for DSS stratified by HPV/p16 status. [Color figure can be viewed at wileyonlinelibrary.com]

We conducted a comparative analysis of treatment outcomes for a cohort of 141 patients (55.1%) with clinical early‐stage tumors (cT1‐2 N0‐1 M0). The results indicated that primary surgical intervention yielded favorable 5‐year PFS rates of 84.7%, OS rates of 74.5%, and DSS rates of 86.8% in this cohort. Multivariable survival analysis demonstrated that primary surgery, in contrast to definitive radiotherapy, was significantly associated with improved PFS (HR = 0.37, *p* = 0.002), OS (HR = 0.55, *p* = 0.012), and DSS (HR = 0.45, *p* = 0.020). A summary of the survival outcomes is presented in Table 3 and Figure 2.

**TABLE 3:** Multivariate analysis for early‐stage (cT1‐2 N0‐1 M0) tumors.

Variable	PFS		OS		DSS	
	HR (95% CI)	*p*	HR (95% CI)	*p*	HR (95% CI)	*p*
Age, y	1.01 (0.98–1.05)	0.457	1.04 (1.01–1.07)	0.008	1.03 (0.99–1.07)	0.141
cT stage						
T1	1		1		1	
T2	3.06 (1.53–6.11)	0.002	1.31 (0.80–2.15)	0.286	2.57 (1.25–5.31)	0.011
cN stage						
N0	1		1		1	
N1	1.56 (0.78–3.13)	0.210	1.55 (0.84–2.84)	0.159	1.64 (0.77–3.49)	0.200
Treatment						
Definitive RT	1		1		1	
Primary surgery	0.37 (0.19–0.69)	0.002	0.55 (0.34–0.87)	0.012	0.45 (0.23–0.88)	0.020

Abbreviations: CI, confidence interval; HR, hazard ratio; RT, radiotherapy.

### 4.3 Oncological Outcomes Concerning HPV/p16 Status

Survival analyses were conducted on patients with known HPV/p16 status. In the univariate analysis, patients who were HPV/p16 positive exhibited survival rates comparable to those who were HPV/p16 negative. The 5‐year PFS, OS, and DSS for patients with HPV/p16 positive disease compared to those with HPV/p16 negative disease were 67.8% versus 66.8% (*p* = 0.662), 72.8% versus 58.4% (*p* = 0.460), and 72.8% versus 72.5% (*p* = 0.839), respectively. The Kaplan–Meier survival curves are presented in Figure 2.

### 4.4 Complications

In total, 19 out of 256 patients (7.4%) experienced severe complications. Among these, one patient developed postoperative pneumonia following surgery and subsequently succumbed to acute respiratory failure. The remaining 18 patients presented with one or more of the following severe complications: permanent feeding tube placement for nutritional support (9 patients), skin ulcer (5 patients), oral mucositis (4 patients), osteoradionecrosis (3 patients), and wound infection after a planned neck dissection that necessitated surgical debridement (1 patient).

## 5 Discussion

There exists a limited number of studies that independently analyze SCCSP from the other oropharyngeal subsites. The current cohort represents one of the largest single‐institution series documented in the literature to date. Our analysis demonstrated that primary is associated with enhanced oncological outcomes for patients with early‐stage SCCSP. Furthermore, our findings did not support the prognostic significance of HPV status in SCCSP.

The baseline patient characteristics observed in this study are largely consistent with those documented in prior research. A significant majority (75.7%) of SCCSPs presented with early T‐stage tumors (T1‐2), which are readily identifiable during clinical examination. The distribution of age, as well as the proportions of smokers, alcohol consumers, patients with cancer history, early T‐stage tumors, and early N‐stage tumors (N0‐1), align closely with findings reported in the existing literature [4, 9, 10, 11, 12]. However, it is noteworthy that our cohort exhibited a higher proportion of male patients, accounting for 91.0%.

It has been well established that patients with HPV‐positive OPSCC typically present with an earlier onset age, improved performance status, reduced tobacco exposure rates, and heightened sensitivity to radiotherapy, resulting in a notably favorable prognosis [2, 13, 14, 15]. However, similar characteristics were not observed in SCCSPs. The prevalence of HPV‐positive disease (28.7%) in SCCSPs in the current study was significantly lower than that found in tonsillar‐related OPSCCs. A summary of the prevalence of HPV/p16 positive disease in SCCSPs reported in the literature is presented in Table 4. In this study, HPV‐positive SCCSPs exhibited comparable onset ages and similar proportions of positive smokers and drinkers when compared to HPV‐negative SCCSPs. Furthermore, our results indicated that HPV status did not hold prognostic significance in SCCSPs. We found that the survival rates of SCCSPs, irrespective of HPV status, were analogous to those of HPV‐negative tonsillar‐related OPSCC [3, 42]. These findings support the assertion that all other OPSCCs (carcinomas arising in the oropharynx outside the tonsils and base of the tongue) should be classified as HPV‐negative OPSCC. Given the considerable research interest in de‐escalating treatment for patients with HPV‐positive OPSCC due to their excellent outcomes, it is imperative to consider the impact of oropharyngeal subsites in treatment planning to mitigate treatment‐related toxicity.

**TABLE 4:** Prevalence of HPV/p16 positive SCCSP.

Author, year	Country	HPV/p16+ tumors	Total number	HPV prevalence	HPV detection
Haeggblom et al., 2017 [6]	Review	59	488	12.1%	unknown
Rietbergen et al., 2013 [16]	Netherlands	9	124	7.3%	p16 IHC and PCR
Tham et al., 2019 [17]	SEER	28	115	24.3%	unknown
Schache et al., 2016 [18]	UK	8	88	9.1%	p16 IHC and PCR or ISH
The present study	China	25	87	28.7%	p16 IHC and PCR
Wang et al., 2016 [19]	China	3	50	6%	PCR
Iyer et al., 2015 [20]	USA	8	46	17.4%	p16 IHC
Lamet al., 2015 [21]	China	3	32	9.4%	PCR
Rietbergen et al., 2012 [22]	Netherlands	0	31	0	p16 IHC and PCR
Nasman et al., 2013 [23]	Sweden	7	22	31.8%	PCR
Lybak et al., 2016 [24]	Norway	5	21	23.8%	PCR
Gillison et al., 2012 [25]	USA	8	20	40%	p16 IHC
Saito et al., 2013 [26]	Japan	3	16	18.7%	p16 IHC
Cerezo et al., 2014 [27]	Spain	5	13	38.5%	p16 IHC
Schache et al., 2013 [28]	UK	4	13	30.8%	PCR
Limbergen et al., 2014 [29]	Belgium	0	11	0	p16 IHC and PCR
Morbini et al., 2014 [30]	Italy	1	10	10%	ISH
Kim et al., 2014 [31]	Korea	4	10	40%	p16 IHC and PCR
Jiang et al., 2015 [32]	USA	0	10	0	ISH
Kim et al., 2015 [33]	South Korea	1	9	11.1%	p16 IHC
Ukpo et al., 2009 [34]	USA	3	8	37.5%	PCR
Park et al., 2012 [35]	Korea	1	8	12.5%	p16 IHC and PCR
McIlwain et al., 2014 [36]	USA	4	6	66.7%	p16 IHC
Davis et al., 2014 [37]	USA	0	6	0	p16 IHC
Grisar et al., 2016 [38]	Belgium	1	5	20%	p16 IHC
Bahl et al., 2014 [39]	India	0	5	0	PCR
Bhosale et al., 2016 [40]	India	0	5	0	p16 IHC
Hoffmann et al., 2012 [41]	Germany	1	4	25%	p16 IHC and PCR or ISH
Total		167	1162	14.4%	

Prior research has demonstrated satisfactory locoregional control rates and cause‐specific survival in patients with SCCSPs who underwent definitive radiotherapy; however, the overall 5‐year OS was approximately 40% [9, 10]. In the current study, the 5‐year OS rate for patients receiving definitive radiotherapy was significantly improved at 52.8%. Furthermore, we observed that patients who underwent salvage surgery exhibited favorable OS and DSS rates. The implementation of salvage surgery may have contributed to the enhanced outcomes observed in our study. Additionally, our findings indicated excellent outcomes for patients treated with primary surgery, with the 5‐year DSS comparable to the results reported by Iyer et al., which included a cohort of 150 patients treated with primary surgical intervention [11]. The 5‐year PFS rate of 52.0% and OS rate of 52.8% for the entire cohort in this study are notably superior to the outcomes reported by Schernberg et al. Notably, a higher proportion (39.8%) of patients in the present cohort received surgical treatment. Given that primary surgery yielded satisfactory outcomes, we hypothesize that the favorable results observed in the primary surgery group may have contributed to the overall improved outcomes for the entire cohort in this study.

The findings of our study hold significant implications for the management of SCCSP. Patients with early‐stage disease exhibit an excellent prognosis when treated with primary surgery. As such, our results may provide a rational basis for reconsidering the management strategies for early‐stage SCCSP, advocating a transition from nonsurgical approaches to transoral robotic surgery (TORS). Existing literature indicates that TORS is associated with enhanced surgical outcomes and improved survival rates, accompanied by a minimal risk of severe complications in the treatment of early‐stage OPSCC [43, 44, 45, 46, 47]. The ORATOR trial, a randomized phase II study, compared primary radiotherapy with primary TORS and neck dissection for early‐stage OPSCC. The findings revealed that while patients receiving radiotherapy demonstrated superior scores on the MD Anderson Dysphagia Inventory (MDADI), the difference decreased with time and never reached a threshold. Importantly, post hoc subgroup analysis of patients with palatine tonsil primaries indicated no significant difference in MDADI scores between those undergoing surgical versus nonsurgical treatment [48, 49, 50]. However, comparative research examining outcomes between SCCSP patients treated with TORS and those receiving non‐TORS therapies remains limited. Given the favorable oncological outcomes associated with TORS and its suitability for early‐stage SCCSP, we propose that TORS may emerge as the preferred treatment modality for early‐stage SCCSP in the future, although further validation is warranted.

There are several limitations due to the retrospective nature of this study. First, a large number of patients lack information about tumor HPV status or p16 expression level. Our analysis of the prognostic role of HPV/p16 status was based on a relatively small sample size; further research with larger and more diverse populations is necessary to establish the reliability and validity of our findings. Second, this study primarily focused on the oncological outcomes of various treatment modalities and did not include functional assessments of swallowing and speech due to insufficient data. Lastly, as a retrospective study, it is inherently subject to the limitations typical of this type of research.

## 6 Conclusions

SCCSPs exhibit characteristics that are more similar to those arising in the oral cavity than to those associated with tonsillar‐related OPSCCs. HPV status does not have prognostic significance for SCCSP. In patients with early‐stage SCCSP, surgery yields a more favorable oncological outcome.

## Conflicts of Interest

The authors declarenoconflicts of interest.

## Data Availability

The data that support the findings of this study are available from the corresponding author upon reasonable request.
